# Optimizing breathlessness management in amyotrophic lateral sclerosis: insights from a comprehensive systematic review

**DOI:** 10.1186/s12904-024-01429-z

**Published:** 2024-04-16

**Authors:** Catarina Bico Filipe, Nuno Reis Carreira, Paulo Reis-Pina

**Affiliations:** 1 Faculty of Medicine, University of Lisbon, Avenida Professor Egas Moniz, Lisboa, 1649-028 Portugal; 2 North Lisboa Hospital Centre, Santa Maria Hospital, Lisboa, Portugal; 3 Bento Menni Palliative Care Unit, Sintra, Portugal

**Keywords:** Amyotrophic lateral sclerosis, Breathlessness, Motor neuron disease, Noninvasive ventilation, Opioids, Palliative care, Systematic review

## Abstract

**Background:**

Breathlessness is a prevalent symptom affecting the quality of life (QOL) of Amyotrophic Lateral Sclerosis (ALS) patients. This systematic review explored the interventions for controlling breathlessness in ALS patients, emphasizing palliative care (PALC), non-invasive ventilation (NIV), opioids, and non-pharmacological strategies.

**Methods:**

A comprehensive search of PubMed, Cochrane Library, and Web of Science databases was conducted. Eligibility criteria encompassed adults with ALS or motor neuron disease experiencing breathlessness. Outcomes included QOL and symptom control. Study designs comprised qualitative studies, cohort studies, and randomized controlled trials.

**Results:**

Eight studies were included, most exhibiting low bias risk, comprising one randomized controlled trial, three cohort studies, two comparative retrospective studies, and two qualitative studies (interviews). Most studies originated from Europe, with one from the United States of America. The participants totaled 3423, with ALS patients constituting 95.6%. PALC consultations significantly improved symptom assessment, advance care planning, and discussions about goals of care. NIV demonstrated efficacy in managing breathlessness, with considerations for device limitations. Opioids were effective, though predominantly studied in non-ALS patients. Non-pharmacological strategies varied in efficacy among patients.

**Conclusion:**

The findings underscore the need for individualized approaches in managing breathlessness in ALS. PALC, NIV, opioids, and non-pharmacological strategies each play a role, with unique considerations. Further research, especially ALS-specific self-management studies, is warranted.

## Introduction

### Rationale

Amyotrophic lateral sclerosis (ALS) is an incurable neurodegenerative disease, characterized by a combination of both upper and lower motor neuron involvement [1].

Respiratory symptoms, especially breathlessness, are common as the disease evolves, and respiratory failure remains the most frequent cause of death, usually within three to five years from when the first symptoms appear [2, 3]. However, more slowly progressive forms of the illness occur in a small proportion of patients [2, 3]. Currently, there is no cure for ALS and no effective treatment to halt or reverse the progression of the disease [3], so the main purpose is to maximize the quality of life (QOL) of ALS patients [4].

According to the *American Thoracic Society*, dyspnea is a term used to characterize a subjective experience of breathing discomfort that consists of qualitatively distinct sensations varying in intensity [5]. In ALS patients, symptom assessment is fundamental.

In ALS, breathlessness occurs due to the progressive weakness of the diaphragm and accessory breathing muscles, retained throat secretions, laryngospasm and aspiration while eating or drinking when there is a predominant bulbar involvement [6]. The relationship between pathology and breathlessness perception is inconsistent, explaining why optimizing disease management alone does not guarantee good symptom control [7].

On that account, breathlessness is a complex symptom that can evoke significant distress.

Descriptors related to air hunger are prominent in the language used by patients to describe their dyspnea [8]. Air hunger is considered the most unpleasant quality of dyspnea, eliciting the strongest emotional response [9]. In a prospective, non-randomized study, the anxiety of choking correlated significantly with the intensity of dyspnea in all patients [10].

High-quality evidence is lacking for most topics in ALS management, and many recommendations provided are based on expert consensus among the working group [11].

As remarkable advances in ALS diagnosis and treatment continue to emerge, there will be an increasing need to support patients and families facing complex decisions amidst significant uncertainty and crucial outcomes for both physical and mental well-being [12]. Palliative care (PALC) involvement from the moment of diagnosis is essential to improve symptom control and QOL for patients with ALS and their families [13, 14]. Patients need assurance that despite having incurable conditions, the chronic symptoms they experience during the progression of the disease will be appropriately alleviated [14].

Considering that: (1) ALS is the most common degenerative motor neuron disorder in adult life; and (2) dyspnea is the symptom that most impairs the QOL in these patients; and (3) no robust review on this theme has been done recently; this study aimed to review the literature on breathlessness control in ALS patients.

### Objectives

This study aimed to systematically review the literature on non-invasive interventions for controlling breathlessness in ALS patients and their effects on (1) overall symptom control and (2) overall QOL.

## Methods

This systematic review followed the recommendations of the *Cochrane Handbook for Systematic Reviews of Interventions* [15], and is reported in accordance with the *Preferred Reporting Items for Systematic Reviews and Meta-Analyses* [16].

### Eligibility criteria

#### Participants

Adults with ALS or motor neuron disease experiencing breathlessness. We included all studies for consideration if they included ALS patients as part of the broader sample population, regardless of the proportion of ALS patients in relation to other participants. Participants from any healthcare setting were eligible.

In regard to breathlessness, we accepted the definitions provided by the authors of the included articles without distinguishing whether it was “air hunger” in general or “dyspnea on minor exertion or talking”, tachypnea, orthopnea, among others.

#### Interventions

PALC, non-invasive ventilation (NIV), pharmacological (opioids) and non-pharmacological treatments specifically targeting breathlessness.

#### Comparators

Any.

#### Outcomes

QOL and symptom control (or burden or management). Any definition and any scales of assessment were accepted.

#### Study design

Any, excluding literature reviews, conference abstracts, book chapters, letters, editorials, commentaries, and academic theses.

We only included articles written in English.

Articles were excluded if they were inaccessible or not subscribed to by our faculty.

### Information sources

PubMed, Cochrane Library, and Web of Science databases were systematically searched for relevant articles. No direct contact with authors was made to identify additional sources.

### Search strategy

The strategy employed was as follows: (“amyotrophic lateral sclerosis” OR “motor neuron disease” OR “ALS” OR “MND”) AND (dyspnea OR breathlessness) AND (“palliative care” OR ”hospice care” OR “terminal care” OR “end of life”). Filters were set for English language and publication dates from 2011 to 2022. The search concluded on January 3, 2023.

### Selection process

The initial screening of articles by title/abstract was performed by the first author. The full text of potentially relevant articles underwent independent eligibility assessments by both authors. Additionally, backward and forward citation searches were conducted, involving the examination of reference lists and the utilization of Scopus and Web of Science to identify articles citing the included studies. Any disagreements related to study selection and data extraction were resolved through discussion and consensus between the authors. To manage references, organize data, and eliminate duplicates, the reference management software EndNote® 20.2.1 for Windows (Clarivate, 2021) was utilized.

### Data collection process

A data extraction form was devised within an Excel 16.0® spreadsheet (Microsoft Corporation, 2023). Both authors independently extracted data from the reports, and the data-charting form underwent review until consensus was achieved for all items. No additional processes were employed for obtaining or confirming data from study investigators.

### Data items

Data were collected for two primary outcomes: QOL and symptom control. All results aligning with each outcome domain in every study were considered, and we included all measures utilized by the study investigators. Results were compiled if they pertained to the interventions specified in our eligibility criteria.

Additionally, data were gathered for various other variables, including authors, country of origin, year of publication, study design, population, interventions, comparator/control groups, main outcomes, and any noteworthy observations.

### Study risk of bias assessment

The appraisal was performed by two independent reviewers. The Joanna Briggs Institute tools were used for qualitative research [17]; and for cohort studies and comparative retrospective chart/registry studies [18]. For randomized controlled trials (RCT), the revised Cochrane RoB2 was employed [19].

### Effect measures

In presenting the synthesis of results, we considered all the effect measures utilized by the original authors for each outcome.

### Synthesis methods

Given the substantial heterogeneity across studies, a meta-regression analysis was omitted, and results are presented in a narrative format. Synthesis involved grouping studies based on eligible interventions.

## Results

### Study selection

**Fig. 1:**
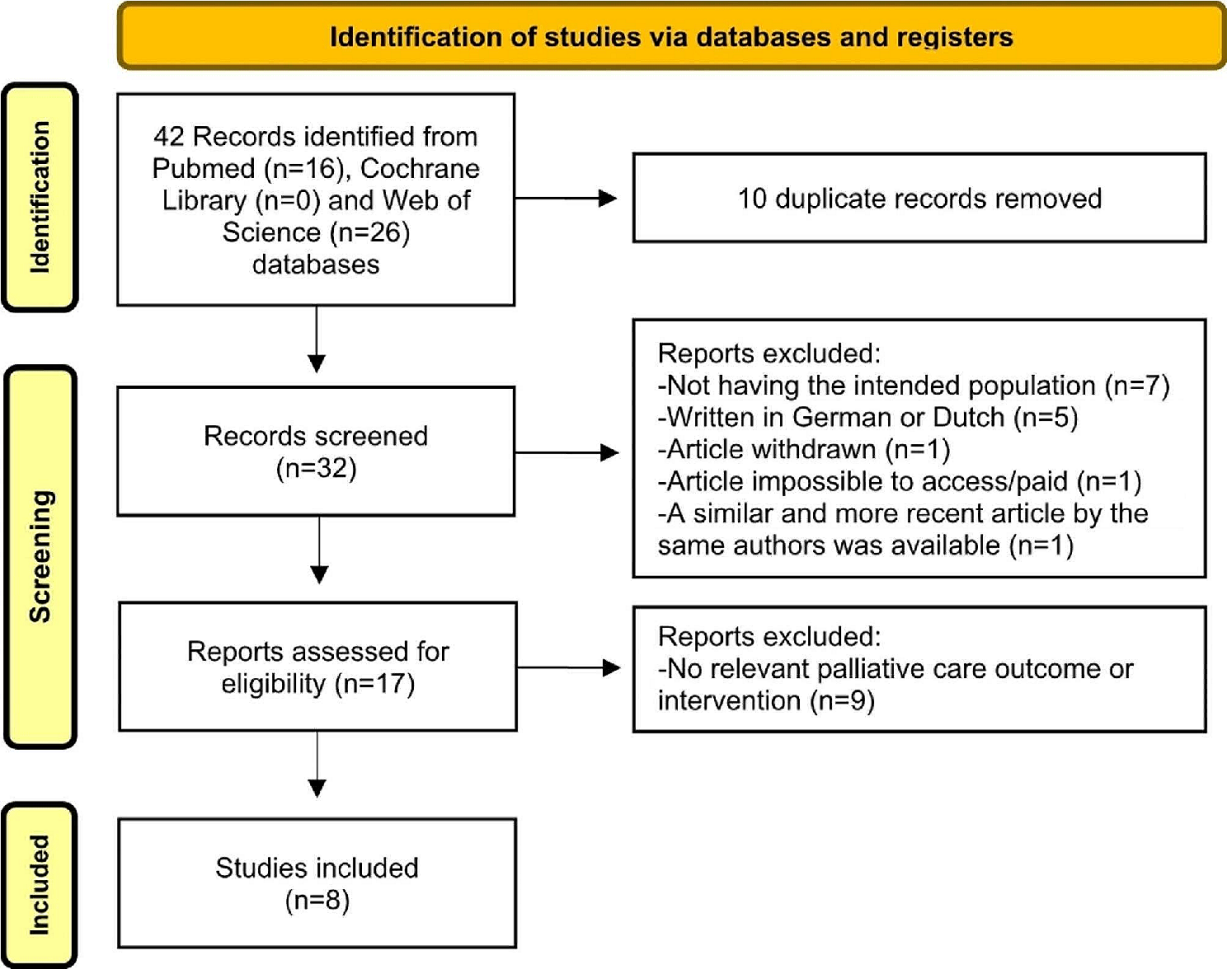
Flow diagram of the study selection process

Forty-two articles were initially identified, and after deduplication, 32 references remained. Exclusions included seven articles not meeting the intended study population criteria (although they included ALS patients, dyspnea was not a specific symptom; instead, patients exhibited constitutional symptoms, tiredness, existential problems, etc.); five in non-English languages; and one inaccessible. Additionally, one article was withdrawn, and another had a more recent counterpart. Screening 17 articles for eligibility resulted in nine exclusions due to irrelevant interventions or outcomes from the PALC perspective (e.g., tracheostomy or invasive ventilation). The systematic review ultimately included eight articles, with the selection process depicted in Fig. 1.

### Study characteristics

Our review included two qualitative studies with interviews [20, 21], one RCT [22], three cohort studies (two retrospective [23, 24], one prospective [25]), and two comparative retrospective chart/registry studies [26, 27]. The majority of articles originated from Europe, with contributions from the United Kingdom [20, 21], Italy [22], Finland [23], Sweden [24, 26], and France [25]. One article was from the United States of America [27]. The total number of participants was 3423, with 95.6% being ALS patients. Four studies included patients with various comorbidities. Detailed characteristics of the studies are presented in Table 1.

**Table 1:** Characteristics of the studies included in the systematic review (*n* = 8)

Study; Country; Year	Study design	Population	Intervention	Comparator/control	Main outcomes	Observations
Gysels MH, et al., UK, 2011	Qualitative study, with a semi-structured interview	48 patients experiencing daily problems of dyspnea (10 ALS, 10 cancer, 10 heart failure, and 18 COPD patients)	None	None	Individual experiences of breathlessness	-A semi-structured interview face-to-face was performed to understand the lived experience of breathlessness. -Nine of the 10 ALS patients were using non-invasive positive pressure ventilation and three of them reported difficulties using it, but for those who had incorporated it into their daily routines, this device was the solution for their daily breathing problems. -Breathlessness affects patients in different ways across different conditions, meaning that its management should be refined and interventions tailored to specific patient groups.
Simon ST, et al., UK, 2016	Qualitative study with Interviews	51 patients suffering from dyspnea (15 chronic heart failure, 14 COPD, 13 lung cancer, 9 ALS) recruited from five outpatient clinics	None	None	Strategies for relieving episodic breathlessness	-The six main strategies described by patients for relieving episodic breathlessness were: reduction of physical exertion, cognitive and psychological strategies, breathing techniques and positions, air and oxygen, drugs and medical devices, environmental and other strategies. -Strategies did not differ between disease groups but between individual patients and were related to the trigger of the dyspnea episode. The identified strategies were largely easy to apply and can be practical aids in the daily care of patients suffering from episodic breathlessness. -Not all recommended strategies (such as leaning forward, breathing techniques, or a draft of cold air) are helpful for all patients demonstrating that any support for patients should be individualized and tailored by the patient’s own experiences.
Veronese S, et al., Italy; 2017	Phase II non-blinded randomized controlled trial – parallel arm design	50 Adult patients severely affected by ALS (*n* = 16), multiple sclerosis (*n* = 18), Parkinson’s disease (*n* = 16). Informal family carers of the patients were also enrolled if they wished.	Immediate referral to PALC service (fast track) (*n* = 25)	16-week wait to referral to the PALC service (standard track) (*n* = 25)	Main outcomes: QOL; Burden of the carers. Secondary outcomes: Physical symptoms, including shortness of breath; social issues; psychological issues; spiritual issues; disability issues.	At baseline, there were no differences between groups. After 16 weeks, fast track participants scored significant improvement in QOL and breathlessness. The mortality was equal in the two arms. Caregiver’s burden was not affected by the service.
Tiirola A, et al., Finland, 2017	Retrospective, longitudinal, cohort study	67 patients with non-malignant disease who died during the period of 2004 to 2013 (32 with ALS)	None	None	Prevalence of symptoms; Prescription of opioids, NIV, and oxygen (comparison between ALS patients and people with other diseases)	-Dyspnea was the most common symptom reported and it increased from admission to the last day of life, but there were no significant differences in the prevalence of symptoms between ALS patients and people with other non-malignant diseases. -During the last 24 h, as-needed opioids were prescribed to nearly all patients (98%), and 75% of them were prescribed regular opioids. -More than one third of patients with ALS used NIV during the last 24 h of life, whereas oxygen was more often given to patients with other diseases.
Morélot-Panzini C, et al., France, 2018	Prospective, longitudinal cohort study	41 ALS adult patients with chronic respiratory failure and indication for NIV	None	None	Effects of NIV on dyspnea	-Patients were allowed to select descriptors of dyspnea in sensory (e.g. “I feel air hunger”; “I am smothering”; “my chest and lungs feel tight or constrictor”; “my breathing requires muscle work or effort”; “my breathing requires mental effort or concentration”; “I am breathing a lot”; “not enough air, smothering or hunger for air”) and affective (depressed, anxious, frustrated, angry) dimensions. -At inclusion 36.6% of the patients considered “lying supine” the worst dyspnoeic episode within the last 15 days, which is bound to have an extremely severe impact on daily life, making normal sleep impossible. But the 27 patients who attended the 1-month follow-up visit and who had used NIV had satisfactory clinical improvement because the most unpleasant breathing episode occurring in the preceding two weeks was “walking a few steps” (25.9%) or “talking or eating” (14.8%). -The positive effects of NIV on dyspnea do not carry over to periods of unassisted breathing, an empirically intuitive notion that has however not been precisely documented before. -This study supports the existence of dissociation between the sensorial and affective dimensions of dyspnea.
Eljas Ahlberg E, et al., Sweden, 2021	Retrospective comparative, registry study	825 patients with ALS as the main cause of death	None	3300 Patients with cancer as the main cause of death	Symptom assessment; Prescription of as-needed drugs; Communication about transition to end of life care.	-Patients with ALS receive poorer end-of-life care than patients dying from cancer, in terms of validated symptom assessments and prescription of as-needed drugs. -About 80% of the patients in both groups had no symptom assessment (other than pain) within the last week of life. -More patients with ALS than with cancer had dyspnea and anxiety in the last week of life. -There was no significant difference in communication about end-of-life between the two groups. -Patients with ALS were less likely to have support from a specialized PALC team than patients with cancer.
Mehta AK, et al., USA, 2021	Retrospective comparative chart review	24 adult ALS patients admitted to two tertiary care academic hospitals from 2013 to 2018	9 patients seen by the inpatient PALC service (PALC group)	15 patients not seen by the inpatient PALC consult team (non PALC group)	Goals of Care; ACP; Symptom control; survival benefit	-This study supports the benefit of inpatient PALC consultations for ALS patients admitted to the hospital for non-elective reasons. -PALC consultations were associated with significantly increased Goals of Care, which includes the discussion of tracheostomy and transition to comfort care, thus ensuring that the patient’s wishes are followed during unexpected, acute changes. -Goals of Care recorded during admission were 89% in the PALC group and 32% in non-PALC group. -The PALC group were more significantly likely to have Goals of Care and ACP forms documented in their medical records at discharge time. -Symptom management was performed by PALC in all 9 patients from the PALC group. Four of these were treated for dyspnea with opioids, benzodiazepines, nebulizers, and supplemental oxygen. Anxiety related to dyspnea was treated with benzodiazepines. -The higher death in the PALC group at the end of the study was not significantly different from the non-PALC group.
Sennfält S, et al., Sweden, 2023 (ahead 2022)	Retrospective cohort study	Main cohort: 93 ALS patients diagnosed in 2016 and followed at the ALS Research Centre, Stockholm, deceased in 2018–2020. Complementary cohort: 2224 ALS patients from all of Sweden deceased in 2011–2020.	None	None	Use of NIV during the last 12 months of life; Prevalence of symptoms	-There was a gradual increase of patients on regular NIV during the last 12 months of life, reaching about 50% at the time of death in both ALS patients with spinal and bulbar onset. -In the week before death, 57 of the 61 ALS patients with anticipated death (death as a culmination of a slow decline) from the main cohort, and 21 of the 29 patients with precipitous death (rapid and unexpected clinical worsening) had dyspnea. For most patients, dyspnea was managed effectively, but in 29.8% of patients from the first group, dyspnea was only partially relieved or not relived at all. -The lower prevalence of dyspnea in the precipitous death group was not statistically significant.

ACP: advance care planning (advanced directive); ALS: amyotrophic lateral sclerosis; COPD: Chronic Obstructive Pulmonary Disease; NIV: non-invasive ventilation; PALC: palliative care; POLST: Physician Order for Life-Sustaining Treatment

Gysels et al., to understand the lived experience of breathlessness, face-to-face interviewed 48 individuals with various diseases: chronic obstructive pulmonary disease (COPD, *n* = 18), heart failure (*n* = 10), ALS (*n* = 10), and cancer (*n* = 10) [20].

Simon et al. interviewed 51 people suffering from dyspnea (15 heart failure, 14 COPD, 13 lung cancer, and 9 ALS) [21]. Patients were invited to talk about their experiences with episodic breathlessness, characteristics and triggers for such episodes, impact on daily living and management strategies [21].

Veronese et al., in a non-blinded RCT-parallel arm study with patients affected by neurodegenerative disease (32% with ALS), compared 25 patients immediately referred to PALC to 25 patients with a 16-week wait for referral [22].

Tiirola et al., in a retrospective cohort study, compared symptom prevalence and the prescription rates of opioids, NIV, and oxygen in 32 ALS patients versus 35 individuals with other diseases [23].

Sennfält et al., in a retrospective study, compared 93 ALS patients deceased in 2018–2020 with a cohort of 2224 ALS patients deceased in 2011–2020, focusing on symptom prevalence and NIV use in the last 12 months of life [24].

In a prospective, longitudinal cohort study, Morélot-Panzini et al. examined 41 ALS adult patients eligible for NIV, who received a visit the day before initiating NIV which was prescribed for eight hours during the night [25]. Participants were prompted to choose descriptors for dyspnea across sensory (e.g., ‘I feel air hunger’) and affective dimensions (feeling depressed, anxious, frustrated, or angry) [25].

In a retrospective registry study, Eljas Ahlberg et al. compared 825 deceased ALS patients to 3300 deceased cancer patients, examining topics such as symptom assessment, prescription of as-needed drugs, and communication about the transition to end-of-life care [26].

Mehta et al., in a retrospective chart review, compared nine ALS patients attended by the inpatient PALC team to 15 ALS patients not seen by the team (non-PALC group), exploring topics including goals of care, advance care planning, symptom control, and survival [27].

### Risk of bias in studies

Among the seven articles reviewed, all were deemed to be of high quality (refer to Table 2).

**Table 2:** Risk of bias for cohort and qualitative studies (*n* = 7)

Study	Critical appraisal tools from the Joanna Briggs Institute	Yes answers	Quality level	Overall appraisal
Gysels MH, et al., 2011	Checklist for Qualitative Research	10	High Quality	Included
Simon ST, et al., 2016		9		
Tiirola A, et al., 2017	Checklist for Cohort Studies	7		
Morélot-Panzini C, et al., 2018		9		
Eljas Ahlberg E, et al., 2021		9		
Mehta AK, et al., 2021		9		
Sennfalt S, et al., 2023, ahead 2022		9		

**Fig. 2:**
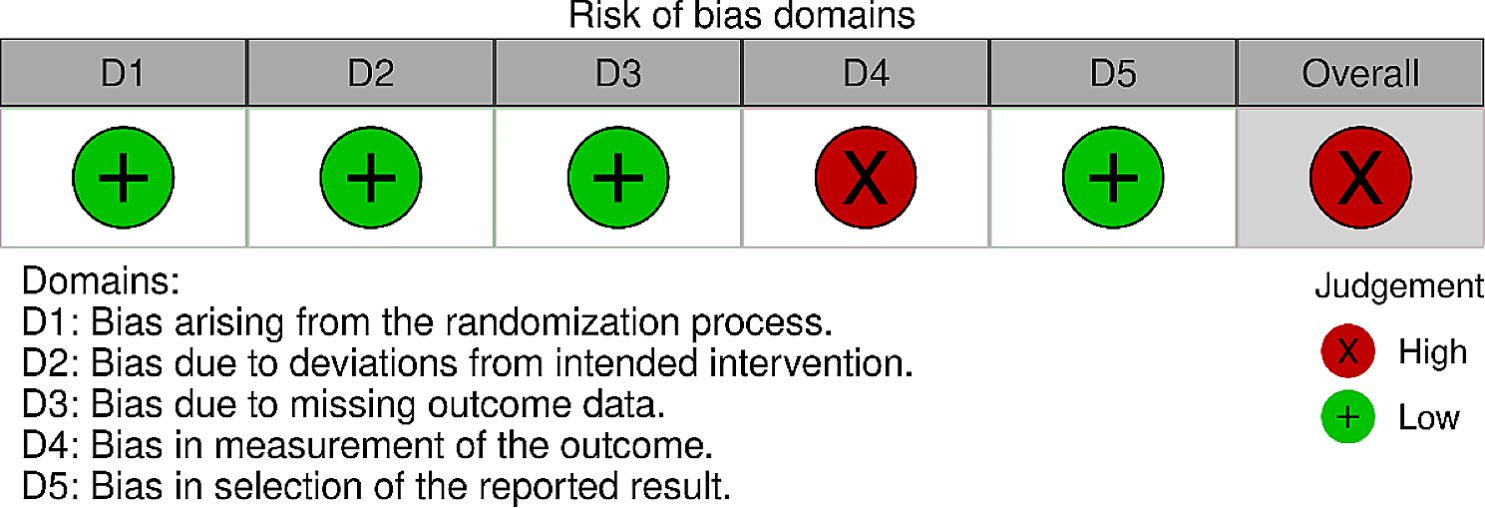
Risk of bias summary for randomized controlled study (*n* = 1)

The RCT conducted by Veronese et al. [22] exhibited bias in outcome measurement (see Fig. 2). In Veronese’s study, there were systematic errors or inaccuracies in the assessment of study outcomes that could lead to distorted results. The authors mentioned that “some tools were not validated” and “only one evaluation and no crossover could be carried out over time, so we do not know if the improvement in the measured domains is maintained.” These issues compromised the reliability, accuracy, and consistency of outcome assessments in this particular study. Despite the overall compromise in quality, given the low risk in the other four domains and the limited research in this area, we opted to include it in our review.

### Results of individual studies

Gysels et al. [20] found that breathlessness varies across conditions, highlighting the need for refined management and tailored interventions for specific patient groups. Nine of 10 ALS patients used positive pressure NIV; however, three reported difficulties [20].

In the study by Simon et al. [21] patients described six main strategies for relieving episodic breathlessness: reducing physical exertion, cognitive and psychological approaches, breathing techniques and positions, air and oxygen, medications and medical devices, and various environmental strategies. The strategies were consistent across disease groups but varied among individuals, often tied to the trigger of the dyspnea episode. These practical strategies, individually applied, can aid in the daily care of patients with episodic breathlessness [21].

In the study by Veronese et al. [22], at the beginning, both groups exhibited similar baseline characteristics; however, after 16 weeks, participants in the fast-track group demonstrated a significant enhancement in both QOL and reduction of breathlessness. Importantly, mortality rates remained comparable between the two arms [22].

Tiirola et al. [23] found that dyspnea, the most common symptom, increased from admission to the last day of life, with no significant differences between ALS patients and those with other non-malignant diseases. Nearly all patients (98%) received as-needed opioids in the last 24 h, and 75% were on regular opioids. Over one-third of ALS patients used NIV in the last 24 h, while patients with other diseases were more frequently given oxygen [23].

Sennfält et al. [24] found that the utilization of regular NIV gradually increased over the last 12 months of life, reaching approximately 50% at the time of death for both ALS patients with spinal and bulbar onset. In the week preceding death, 57 out of 61 ALS patients with anticipated death (a culmination of a slow decline) and 21 out of 29 patients with precipitous death (rapid and unexpected clinical worsening) experienced dyspnea. While dyspnea was effectively managed for most patients, around 29.8% in the “anticipated death” group found only partial or no relief. The lower prevalence of dyspnea in the precipitous death group did not reach statistical significance [24].

Morélot-Panzini et al. [25] found that 36.6% of patients considered ‘lying supine’ as the most severe dyspneic episode within the last 15 days, severely impacting daily life and sleep. However, at the 1-month follow-up, the 27 patients using NIV reported improved episodes, with ‘walking a few steps’ (25.9%) or ‘talking or eating’ (14.8%) as the most unpleasant breathing experiences. NIV initiation significantly reduced sensory dyspnea descriptors but had no significant impact on affective descriptors [25].

Eljas Ahlberg and Axelsson discovered that eight out of ten patients, whether with cancer or ALS, did not undergo symptom assessment (other than pain) within the last week of life [26]. Despite this, 93.3% of ALS patients could communicate less than a week before death, making symptom assessment, including dyspnea, feasible. More ALS patients experienced dyspnea and anxiety compared to cancer patients in the final week. The study found no significant difference in end-of-life communication between the two groups [26].

Mehta et al.‘s study highlights the positive impact of inpatient PALC consultations on ALS patients admitted for non-elective reasons [27]. PALC consultants assessed at least one physical symptom, even when the initial consultation request did not explicitly mention symptom management. The PALC group showed a significant increase in documented Goals of Care compared to the non-PALC group, with 89% and 32% respectively during admission. Moreover, the PALC group had a higher likelihood of having Goals of Care and Advance Care Planning forms documented in their medical records at discharge [27].

### Results of synthesis

Most of the articles included were about PALC and NIV in ALS patients.

#### Palliative care in amyotrophic lateral sclerosis

Patients with ALS were observed to receive less support from specialized PALC teams during the final week of life compared to cancer patients [26]. This discrepancy underscores a potential gap in end-of-life care, as individuals with ALS may experience suboptimal support, characterized by limited validated symptom assessments and prescriptions for as-needed drugs [26]. This situation highlights the necessity for educational interventions aimed at enhancing symptom assessments within PALC, ultimately elevating the quality of care provided to ALS patients.

Notably, PALC consultations played a vital role in improving end-of-life discussions, specifically concerning Goals of Care. Topics such as tracheostomy and the transition to comfort care were significantly enhanced through these consultations [27]. This is particularly crucial considering that, during the end-of-life stage, patients often rely on their families and healthcare providers to communicate their medical wishes [27]. However, a common challenge arises when there is a disparity between patients’ preferences and the medical interventions received, especially in situations where direct communication is not possible. PALC consultations act as a valuable mechanism to ensure alignment with the patient’s preferences, even amid unforeseen acute changes [27].

Despite reservations about the quality of the included RCT, its findings suggest that the involvement of a specialized PALC team positively influences the QOL of patients with neurodegenerative conditions, including ALS [22]. The multifaceted role of the PALC team extends beyond symptom assessment to encompass prescribing medications, providing nursing care, facilitating physical therapies, and offering psychological support [22]. Importantly, it’s noted that the support of a PALC team does not hasten death, emphasizing the beneficial aspects of PALC in improving the overall well-being of individuals facing neurodegenerative conditions like ALS [22].

#### Non-invasive ventilation in amyotrophic lateral sclerosis

The use of NIV demonstrated effectiveness in managing daily breathing problems when incorporated into the daily routines of ALS patients [20]. As the disease progresses, there is an observed increase in the prevalence of NIV use among ALS patients [24]. In the final stages of the disease, specifically during the last 24 h of life, one-third of ALS patients were reported to have utilized NIV [23].

Research indicates that NIV has positive effects on sensory dyspnea during assisted breathing in ALS patients. However, it is important to note a potential dissociation between the sensory and affective dimensions of dyspnea in this context [25]. This observation emphasizes the nuanced impact of NIV on different aspects of the breathing experience for ALS patients.

#### Opioid use in amyotrophic lateral sclerosis

Opioids have been employed to manage breathlessness in ALS patients towards the end of life, with notable patterns observed a significant majority of non-cancer ALS patients received as-needed opioids in the last 24 h before death [23]. However, only three out of four ALS patients had regular prescriptions for opioids to address breathlessness [23]. Additionally, less than half of the ALS patient population received opioid treatment specifically for dyspnea [27].

These findings underscore the variability in opioid utilization among ALS patients and highlight potential areas for improvement in the management of breathlessness in this context.

#### Non-pharmacological treatment in amyotrophic lateral sclerosis

Participants employed various strategies to relieve episodic breathlessness, including reducing activity, changing positions (standing still, sitting, lying down), cognitive techniques (concentration, positive thinking), distraction methods, and the use of breathing techniques (such as, lip breathing, abdominal breathing, leaning forward, or putting the arms up) [21]. Positions for relief varied, with preferences for lying down, standing up, or using pillows. Fresh air, cold temperature, and moistening the mouth were commonly cited as helpful. Some participants found relief in chewing gum or consuming refreshing food [21].

The diversity of effective strategies highlights the importance of individualized support for each patient’s experience.

## Discussion

### Summary of evidence

In our systematic review (*n* = 8), we observed that: (1) PALC consultations enhance symptom assessment and control, foster more comprehensive discussions and documentation of goals of care, and advance advanced care planning for ALS patients; (2) NIV effectively manages breathlessness in ALS patients; (3) Opioids prove effective and safe for breathlessness, although most studies are conducted in COPD and cancer patients; (4) The efficacy of non-pharmacological dyspnea control strategies varies among patients with the same disease, emphasizing the need for individualized approaches.

### Palliative care in amyotrophic lateral sclerosis

PALC in ALS embraces a comprehensive strategy addressing various dimensions of breathlessness. Physical symptoms necessitate thorough differential diagnosis, along with pharmacological and non-pharmacological management, as well as regular review [28]. The breathing–thinking–functioning model delineates how breathlessness intertwines with psychological, physiological, social, and behavioral factors, perpetuating a cycle that worsens the symptom [7]. This model underscores the significance of Breathlessness Services incorporating tailored support for patients with respiratory diseases, acknowledging the interconnected impact on breathing patterns, mental well-being, and physical functioning [7]. Proactive assessment of physical and psychosocial issues is recommended to reduce the intensity, frequency and need for crisis intervention (unplanned care) [28].

A Consensus Document from the *European Association for Palliative Care* and the *European Academy of Neurology* has emphasized the role of PALC for all neurological diseases [28]. Studies on PALC have demonstrated improvements in QOL, symptoms, patient and family satisfaction [28]. PALC therapeutic interventions are hypothesized to indirectly increase survival duration [29].

Conversations about advance care planning [12], and psychosocial and spiritual issues [30] should be initiated early in the disease or whenever the patient inquires. Ongoing discussions about goals of care should be part of routine ALS follow-up [11]. Careful discussion about the wishes of the patient and family – including place of death, funeral arrangements, will, etc. – may be important to ensure that all parties are as prepared as possible [28].

No matter the path, a multidisciplinary PALC approach emphasizing support of patient and caregiver decisions coupled with early open conversations about EOL issues provides patients with dignity in death [1]. The PALC and EOL measure set by the *National Quality Forum* includes measures within the following domains: comfortable dying, symptom screening, beliefs and documentation of values, care preferences, and treatment preferences [31]. The issues of dying and death should be discussed with patients, particularly as interventions such as NIV and gastrostomy are introduced [32].

### Non-invasive ventilation in amyotrophic lateral sclerosis

NIV may improve respiration, QOL [33], and survival duration [29, 33]. Depending on individualized respiratory function, patients must use NIV for prolonged periods of time, ranging from 8 h/day (overnight while sleeping) up to 24 h/day [29]. Patients may initially require slightly longer time to adjust to adaptive ventilator modes, adherence is similar over the longer term [34].

In ALS patients without severe bulbar dysfunction, NIV improved survival (median benefit of 205 days) in a RCT, with maintenance of, and improvement in, QOL [33]. In patients with severe bulbar impairment, NIV improved sleep-related symptoms, but was unlikely to confer a large survival advantage [33]. Moderate-quality evidence from a single RCT also showed that survival and QOL were significantly improved in the subgroup of people with better bulbar function, but not in those with severe bulbar impairment [35].

Recent advancements in NIV technology have led to the development of more sophisticated delivery systems [1]. These devices offer various modes such as bilevel, volume- or pressure-controlled breath delivery, either at specific times or coordinated with spontaneous efforts. Alternatively, they may employ threshold ventilation through adjustable, volume-assured pressure support [34]. Adaptive ventilator settings enable the device to detect and compensate for lapses in baseline ventilation, even in cases of progressive respiratory weakness [34].

Current guidelines primarily focus on NIV initiation and may neglect psychosocial considerations [36]. Optimizing NIV utilization in ALS/MND patients demands a holistic strategy, encompassing specialized multidisciplinary care, patient and family education, caregiver involvement in decision-making, and more. Supportive interventions, consistent monitoring, and continual discussion of patient preferences are equally crucial [36].

The discussion on NIV in ALS requires a nuanced approach based on disease stage [32]. While NIV helps alleviate respiratory symptoms, acknowledging limitations such as potential dependence and challenges like nasal bridge ulceration during continuous use is crucial [37]. Moreover, NIV may reduce overall patient comfort, impede communication, and hinder mobility [29]. Exploring mitigation measures like hydrocolloid dressings and alternate mask systems is crucial. Strategies such as switching between traditional nasal masks and nasal cushion systems, using a mouthpiece during wakefulness (requiring some facial muscle strength), and considering intermittent abdominal pressure ventilation or cuirass ventilators can aid patients [37]. Challenges such as asynchrony and difficulties in mask application, particularly in patients with upper limb involvement, require attention [6, 37]. Acknowledging these aspects ensures informed decision-making regarding the duration and feasibility of NIV use. Recent studies indicate that the benefits of NIV, including increased patient survival and improved mood, outweigh perceived QOL negatives [29].

Considering the close relationship between NIV and oxygen, it is imperative to briefly address the issue of “oxygen need.” Oxygen use should be approached with caution in dyspneic ALS patients, as it may potentially exacerbate hypercapnia [38]. Despite the study by Simon et al. mentioning that ALS patients used oxygen as a strategy to relieve episodic breathlessness [21], and Tiirola et al. finding that oxygen prescription is used in treating dyspnea in ALS patients (albeit less frequently than in individuals with other diseases) [23], oxygen should be regarded as a pharmacological agent in hypoxemic patients and not prescribed based solely on intuitive assumptions of its benefits. Moreover, there is no need to obtain oxygen if this increases the complexity of the end­of­life care plan [38].

Healthcare professionals should be prepared for the discontinuation of NIV at the end of life [32], either at the request of a cognitively capable patient or as part of advance care planning, whether through a living will, power of attorney, or advance directive. In such situations, anticipation and effective management of breathlessness and distress symptoms are paramount. The Association for Palliative Medicine of Great Britain and Ireland has issued a comprehensive professional guideline on the withdrawal of long-term ventilation in ALS patients [38]. This guideline elaborates on PALC concepts such as sedation and enhanced symptom control.

### Opioid use in amyotrophic lateral sclerosis

In cases where NIV is declined or insufficient, opioids emerge as a safe and effective option for improving dyspnea in ALS patients [13, 39]. Given the overlap in cortical structures activated by pain and air hunger, it is reasonable to consider whether opiates affect central pathways involved in air hunger perception [40] In a blinded RCT with six healthy volunteers, a moderate morphine dose notably relieved laboratory dyspnea, with a minor impact on ventilation. This model established a significant treatment effect, consistent with clinical opioid studies [41]. In managing dyspnea, opioids’ therapeutic effect is enhanced by reducing overall oxygen consumption through alleviating anxiety, fear, and panic [10]. Additionally, decreased respiratory effort reduces oxygen consumption of the respiratory muscles [10].

While existing studies predominantly focus on COPD or cancer patients [13], extrapolating these findings to ALS patients is reasonable.

Advancements in applying PALC to refractory neurological diseases have showcased the efficacy of opioids in ALS [10, 14, 30, 42]. Opioids, as symptomatic treatments for dyspnea, should be readily accessible, even early in the disease progression [43]. Several studies have documented opioid use in ALS patients for controlling breathlessness.

In a prospective study of six bulbar ALS patients in a PALC unit, oral morphine was administered (initial dose: 6.3 ± 7.0 mg), with titration possible in 1 mg increments if needed [10]. Results demonstrated a significant reduction in respiratory rate and dyspnea intensity 120 min post-morphine administration. No significant changes were observed in transcutaneous carbon dioxide partial pressure or oxygen saturation, and oxygen insufflation did not notably decrease dyspnea intensity. Respiratory depression cases were not reported [10].

In a retrospective case-based analysis of 84 ALS patients until death, morphine was administered to all dyspneic patients, primarily orally or via gastrostomy [44]. Most patients (69.9%) did not use mechanical ventilation (MV) until death (no-MV group), while 22.9% utilized only NIV. The final dosage equivalent of morphine in the NIV group was significantly higher (mean 65.7 ± 54.6 mg, range 10–200 mg) than in the no-MV group (mean 31.7 ± 26.9 mg, range 0–120 mg). Additionally, opioid use duration in months was significantly longer in the NIV group compared to the no-MV group (8.37 ± 8.09 vs. 2.70 ± 3.17) [44].

Oral opioid administration is convenient but requires titration due to wide individual variability in enteral drug bioavailability. Studies in ALS patients with breathlessness typically use doses averaging 30 mg or less [10, 45–47], with some reaching up to 200 mg [44], or even 520 mg [48]. Most studies demonstrate opioid efficacy in managing breathlessness with manageable adverse effects, primarily obstipation [45]. Titrating dosages against clinical symptoms rarely leads to life-threatening respiratory depression [14, 49]. A systematic review and meta-analysis (*n* = 67 studies) found no evidence of significant or clinically relevant respiratory adverse effects of opioids for chronic breathlessness [50].

The potential applicability of opioids in ALS merits further investigation.

### Non-pharmacological treatment in amyotrophic lateral sclerosis

In our systematic review no studies have explored the effectiveness of self-management strategies in mitigating breathlessness among ALS patients. A recent Cochrane review, encompassing 27 studies and 6008 participants with COPD, revealed that self-management interventions correlate with enhanced QOL, reduced likelihood of respiratory-related hospital admissions, and a low risk of harm [51]. However, the current lack of ALS-specific studies emphasizes the need for dedicated research to explore the efficacy of self-management strategies in enhancing the QOL of individuals with ALS experiencing breathlessness.

In conclusion, the multifaceted nature of breathlessness in ALS necessitates a holistic and personalized approach, integrating pharmacological and non-pharmacological interventions. As Creutzfeldt and Kluger stated, neuropalliative care is an emerging field with a bright future [12], but further research, especially in the context of ALS, is essential to enhance the understanding and implementation of effective strategies to improve the overall well-being of these patients.

## Limitations

This study has several limitations. Firstly, it was not registered in a systematic review database. Secondly, despite some studies in our search ostensibly targeting ALS patients, a detailed analysis revealed their exclusion, resulting in the elimination of several articles.

Additionally, certain studies encompassed varied patient groups, making it unclear whether the described interventions were specifically tailored for ALS patients. The majority of the incorporated studies were retrospective, comprising two cohort studies and two chart or registry-based studies. Qualitative studies, though diverse, had limited representation of ALS patients (only 19 participants in two studies). Consequently, the findings may not be readily generalizable.

Regarding NIV interventions, we did not collect data on types of devices, timings of initiation, use duration, modes of ventilation, settings, etc., which would have been useful for comparing results according to our outcomes.

## Conclusions

This systematic review offers insights into the multifaceted management of breathlessness in ALS; however, there is still limited available evidence on the optimal management of dyspnea in ALS. PALC consultations emerge as instrumental in enhancing symptom control, advanced care planning, and discussions about goals of care. NIV proves effective but requires a nuanced approach considering device limitations. Opioids, while effective, lack extensive ALS-specific studies. Non-pharmacological strategies exhibit varying efficacy, emphasizing the need for personalized approaches.

Notwithstanding the study’s limitations, notably the lack of ALS-specific self-management studies and the prevalence of retrospective designs, it accentuates the imperative for additional research. Integrating the insights gleaned from this review, in conjunction with sustained research endeavors and the advocacy for educational interventions, will foster a refined and personalized approach, ultimately enhancing the overall well-being of ALS patients grappling with breathlessness.

## Data Availability

No datasets were generated or analysed during the current study.

## Abbreviations

ALS: amyotrophic lateral sclerosis
COPD: Chronic Obstructive Pulmonary Disease
NIV: non-invasive ventilation
PALC: palliative care
QOL: quality-of-life
RCT: randomized controlled trial
